# Commentary: Efgartigimod for generalized myasthenia gravis: a comprehensive review of clinical evidence and future perspectives

**DOI:** 10.3389/fneur.2026.1975630

**Published:** 2026-10-16

**Authors:** Ana Belén Fernández

**Affiliations:** Anesthesia and Postoperativel Intensive Care Department, Nuestra Sra De Candelaria University Hospital, Santa Cruz de Tenerife, Spain

**Keywords:** abdominal sepsis, early immunotherapy, efgartigimod, FcRn antagonist, myasthenic crisis, post-operative complications

I read with great interest the comprehensive review by Jiang et al. (1), which offers a timely and well-structured synthesis of the clinical evidence supporting efgartigimod in generalized myasthenia gravis (gMG). I wish to open this commentary by directing attention to a less discussed situation: post-operative respiratory failure in a patient with poorly controlled myasthenia gravis, where myasthenic and/or cholinergic crisis should not be treated as a diagnosis of exclusion, but as a condition warranting immediate priority intervention once clinically suspected. In clinical practice, however, respiratory failure in this population is often labeled as multifactorial—attributed to sepsis, fluid overload, or post-operative deconditioning—rather than recognized as myasthenic crisis; this diagnostic ambiguity, rather than uncertainty that crisis belongs in the differential diagnosis, is what merits closer attention. The review by Jiang et al. (1) offers a valuable synthesis supporting early intervention, from the pivotal ADAPT trial through to real-world experience, and the authors are to be commended for consolidating a rapidly expanding literature, particularly on thymoma-associated disease and perioperative thymectomy management.

However, I wish to highlight a gap that I believe merits further discussion: the lack of specific evidence addressing the post-operative setting, particularly following urgent abdominal surgery in these immunosuppressed patients, a situation likely to become increasingly common as more patients with difficult-to-control gMG are maintained on biologic therapy. The reviewed evidence largely addresses elective surgery, without developing the perspective of urgent abdominal surgery and severe sepsis, such as perforated diverticulitis (1). Several additional mechanisms may compound the risk of crisis in this population, summarized in Figure 1: masking of clinical and septic signs by immunosuppression, infection-driven autoantibody production (7), nutritional deficits from prolonged fasting—including hypophosphatemia, which directly impairs diaphragmatic contractility and is further favored by refeeding syndrome (9, 12)—and sepsis-related oxidative stress affecting the motor endplate (10, 11).

**Figure 1:**
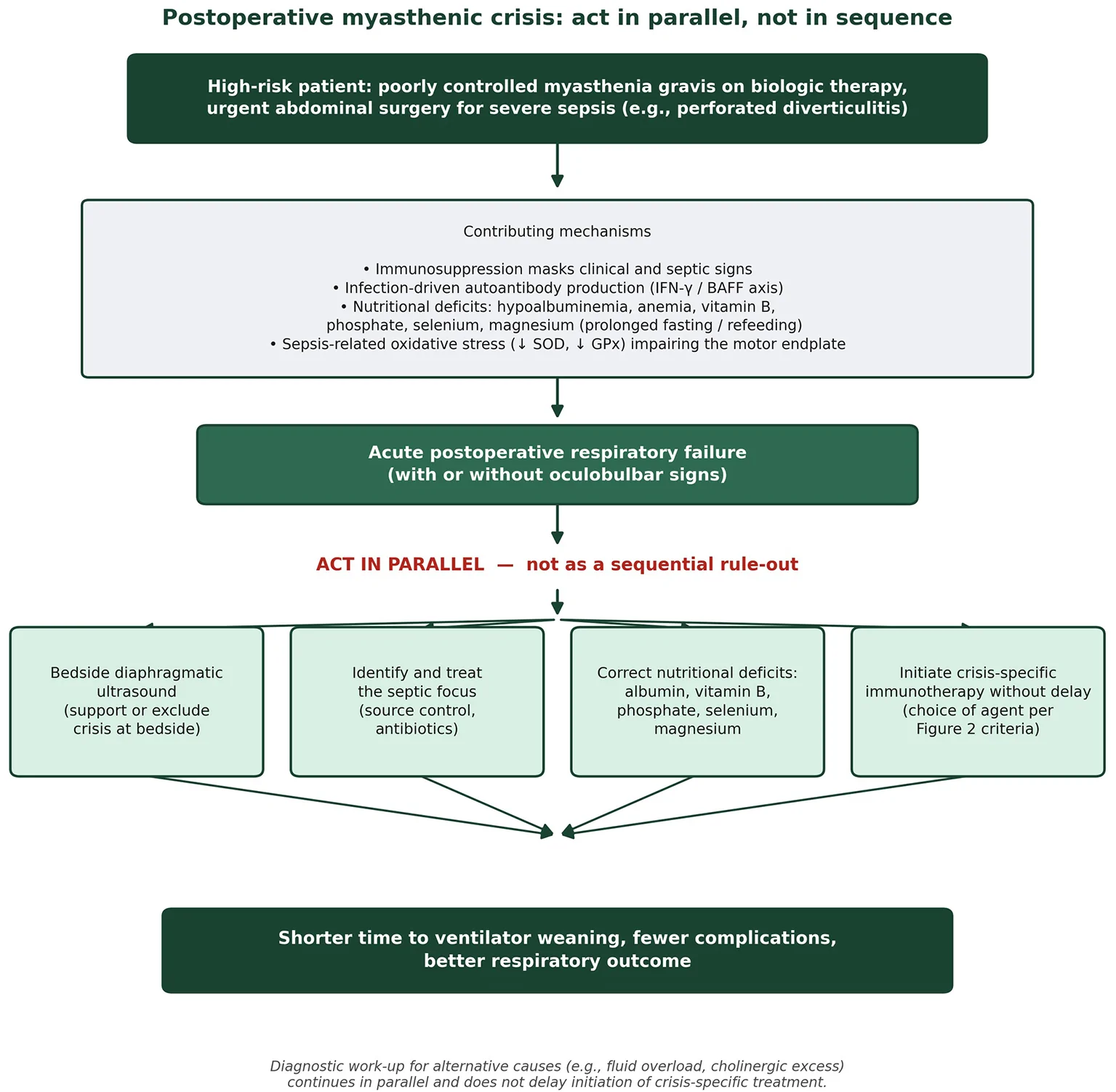
Suggested parallel-action approach to acute respiratory failure in patients with poorly controlled myasthenia gravis on biologic therapy undergoing urgent abdominal surgery. FcRn, neonatal Fc receptor; PLEX, plasma exchange.

The review appropriately highlights that myasthenic crisis is a life-threatening complication requiring rapid antibody reduction, citing successful rescue with efgartigimod, mostly in thymectomy-related settings. Little specific guidance exists on crisis precipitated by intra-abdominal sepsis after emergency laparotomy, a scenario with distinct confounding factors: post-operative sedation and pain mask oculobulbar signs; ileus introduces uncertainty regarding pyridostigmine absorption, complicating the distinction between myasthenic and cholinergic crisis; and biologic therapy raises the question, addressed below, of whether plasma exchange (PLEX) remains appropriate as first-line rescue in these patients.

A further point I consider underrepresented concerns clinical presentation. The review, in line with most of the literature it cites, assumes that oculobulbar findings will accompany or precede respiratory failure, prompting early suspicion of crisis. In my clinical experience, myasthenic crisis in the critically ill post-operative patient can present as isolated hypercapnic respiratory failure, without any oculobulbar signs. In the intubated patient on mechanical ventilation (MV), where formal cooperation-dependent pulmonary function testing is not feasible, bedside diaphragmatic ultrasonography (2, 3) offers a rapid, repeatable bedside tool whose findings can support or exclude the suspicion of crisis, avoiding unnecessary delays in management, and deserves more explicit consideration in future guidance on myasthenic crisis recognition in post-operative intensive care units (PICU).

But I would like to raise what I consider the central clinical dilemma: the appropriate sequencing of exclusion vs. treatment (Figure 1). A recurring pattern is the instinct to sequentially rule out competing explanations for respiratory failure—fluid overload, uncontrolled sepsis, cholinergic excess, mechanical or pulmonary causes—before committing to crisis-specific immunotherapy. Pure fluid overload rarely accounts on its own for the need for intubation: one would expect a response to diuretics and non-invasive ventilation (NIV), together with a distinct lung ultrasound pattern (8), and the two are not mutually exclusive, since respiratory muscle weakness can amplify even mild fluid overload. The correct approach is to address possible crisis in parallel, not after a congestive cause is fully excluded. In a patient with poorly controlled gMG on biologic therapy and acute hypercapnic respiratory failure of neuromuscular pattern in the early post-operative period, the pretest probability of crisis is high enough that treatment should start in parallel with, rather than subordinate to, exclusion of alternative diagnoses. Tang et al. (4) describe consistent improvement with efgartigimod across 20 patients with myasthenic crisis, Song et al. (5) report ventilator weaning within a mean of 10.4 days, and absence of MG-specific immunosuppressive treatment at ICU admission is independently associated with prolonged weaning (6); together, these findings support initiating specific treatment without delay.

The question of PLEX vs. FcRn antagonism in patients already on biologic therapy deserves nuance rather than a straightforward recommendation of one over the other. Efgartigimod itself, by inhibiting FcRn, non-specifically reduces circulating IgG, which may also accelerate clearance of IgG-based therapeutic monoclonal antibodies; it should not be assumed that efgartigimod better preserves concomitant biologic therapy than PLEX. Biologic agents in gMG are also not homogeneous: B-cell-depleting agents such as rituximab differ mechanistically from complement inhibitors such as eculizumab or ravulizumab. For rituximab, PLEX matters most shortly after infusion; once B-cell depletion is established, the pharmacodynamic effect may persist despite falling circulating levels. Complement inhibitors, by contrast, depend on continuous plasma concentrations, so lowering circulating levels—by PLEX or FcRn inhibition alike—could more directly compromise efficacy. This does not imply that efgartigimod is established as equivalent or superior to PLEX or intravenous immunoglobulin in myasthenic crisis, where supporting evidence for efgartigimod remains limited to case series; the point is rather that crisis-specific immunotherapy should not be delayed pending exclusion of alternative diagnoses. Yet in a patient with intra-abdominal sepsis after emergency laparotomy, each option carries a risk profile overlapping with the condition treated, warranting explicit criteria rather than a general statement of individualization. Figure 2 proposes a decision framework based on hemodynamic and coagulation status, sepsis control, renal and volume status, and the specific biologic agent, since no single dimension alone determines the safest choice.

**Figure 2:**
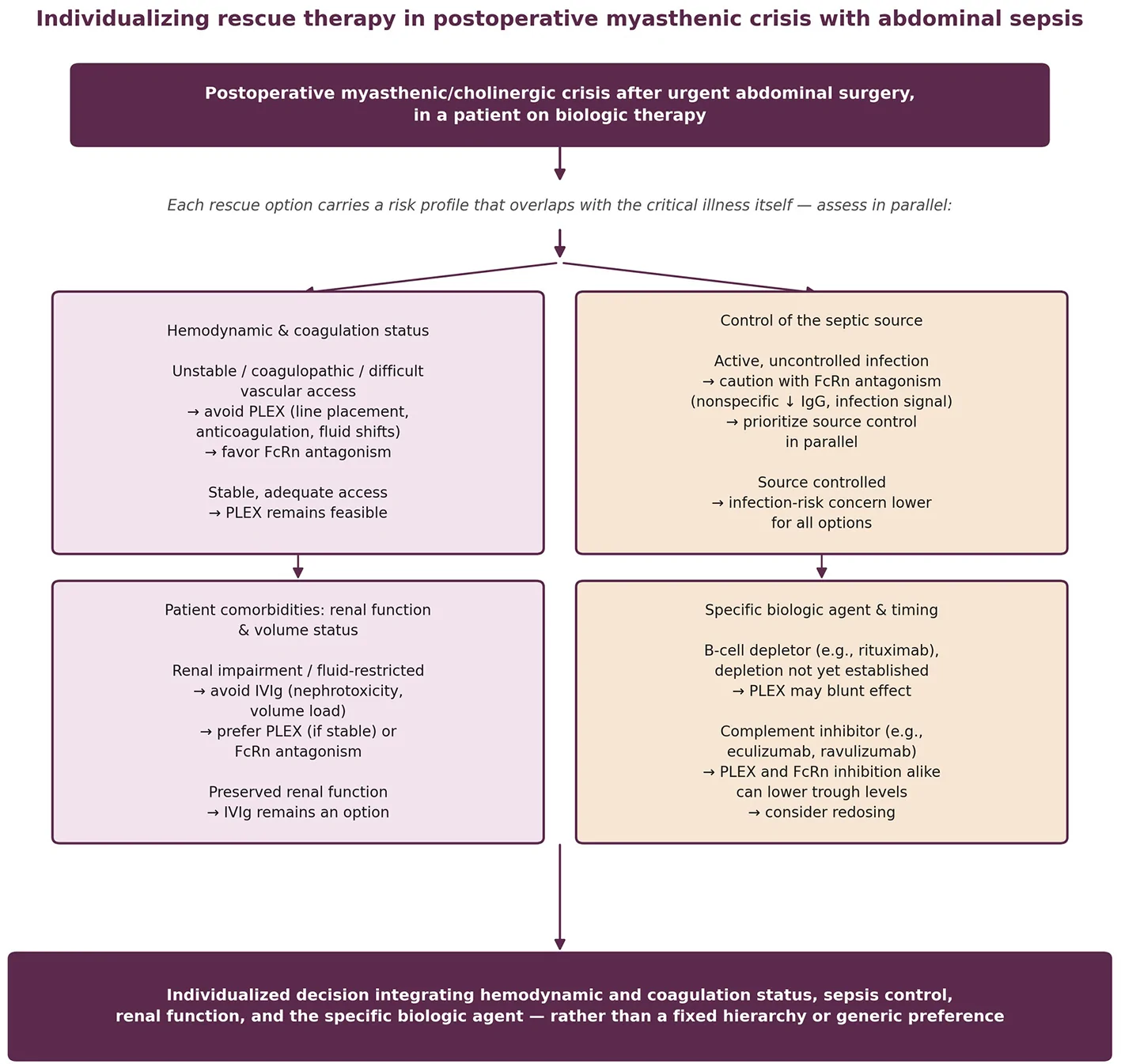
Proposed decision framework for individualizing rescue therapy (plasma exchange, intravenous immunoglobulin, or FcRn antagonism) in post-operative myasthenic crisis complicated by abdominal sepsis, based on hemodynamic and coagulation status, control of the septic source, renal function and volume status, and the specific biologic agent and its mechanism. FcRn, neonatal Fc receptor; IVIg, intravenous immunoglobulin; PLEX, plasma exchange.

I propose that future guidance address, more explicitly, the recognition of myasthenic crisis in patients whose respiratory failure is otherwise labeled as multifactorial after urgent abdominal surgery, the role of diaphragmatic ultrasonography in atypical presentations lacking oculobulbar signs, and the need to individualize rescue therapy by biologic agent, mechanism, and timing, without implying superiority of any single modality in myasthenic crisis.
